# *oriD* structure controls RepD initiation during rolling-circle replication

**DOI:** 10.1038/s41598-017-18817-6

**Published:** 2018-01-19

**Authors:** Algirdas Toleikis, Martin R. Webb, Justin E. Molloy

**Affiliations:** 1 The Francis Crick Institute, 1 Midland Road, London, NW1 1AT UK; 2 WMS - Cell and Development Biology, University of Warwick, Coventry, CV4 7AL UK

## Abstract

Bacterial antibiotic resistance is often carried by circular DNA plasmids that are copied separately from the genomic DNA and can be passed to other bacteria, spreading the resistance. The chloramphenicol-resistance plasmid pC221 from *Staphylococcus aureus* is duplicated by a process called asymmetric rolling circle replication. It is not fully understood how the replication process is regulated but its initiation requires a plasmid-encoded protein called RepD that nicks one strand of the parent plasmid at the double-stranded origin of replication (*oriD*). Using magnetic tweezers to control the DNA linking number we found RepD nicking occurred only when DNA was negatively supercoiled and that binding of a non-nicking mutant (RepDY188F) stabilized secondary structure formation at *oriD*. Quenched-flow experiments showed the inverted complementary repeat sequence, ICRII, within *oriD* was most important for rapid nicking of intact plasmids. Our results show that cruciform formation at *oriD* is an important control for initiation of plasmid replication.

## Introduction

**Figure 1:**
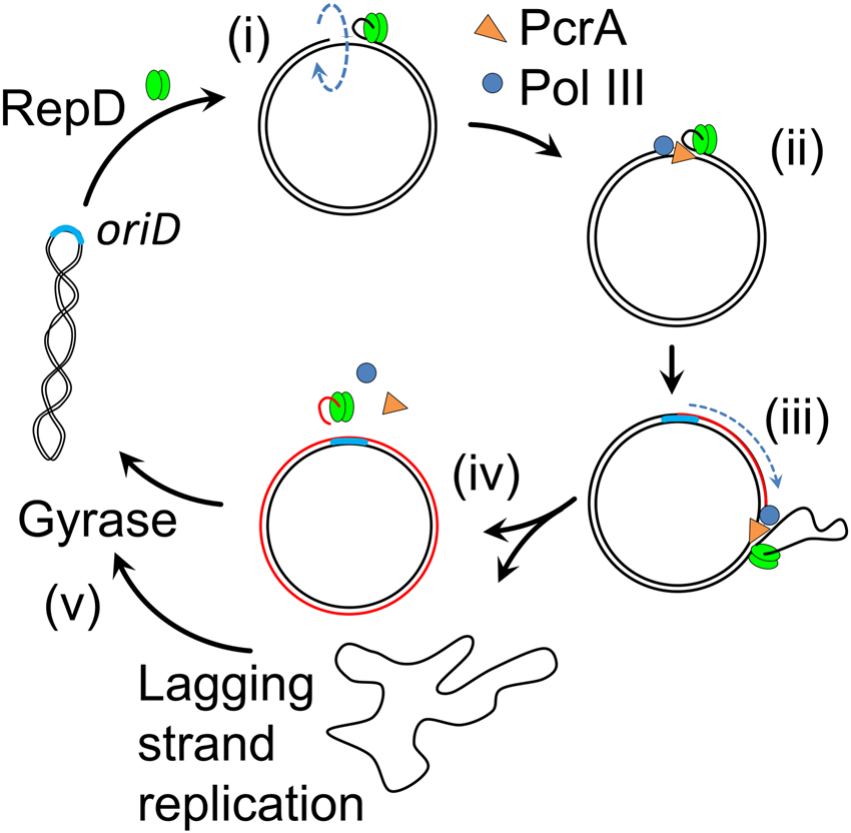
The rolling circle plasmid replication **(i)** The initiator protein (RepD, green) nicks DNA at the site of double-stranded origin *oriD* making a covalent complex with DNA. **(ii** & **iii)** PcrA helicase (orange) binds the exposed section of single-stranded DNA, unwinding the parent plasmid DNA allowing DNA polymerase III (blue) to commence synthesis of a new DNA strand (red strand). **(iv)** After DNA synthesis has proceeded all the way around the plasmid, reaching the region of *oriD*, replication is terminated, RepD is released along with the single-stranded DNA which is then replicated by another round of DNA synthesis. **(v)** Finally, the two newly synthesized daughter plasmids are supercoiled by DNA gyrase so that further rounds of replication may proceed.

Mechanisms that control initiation of DNA replication are essential to ensure DNA synthesis occurs at the correct time and at the correct level in order to maintain the genetic integrity of the organism^1^. The pT181 family of plasmids contain antibiotic resistance genes and replicate by a mechanism known as rolling circle replication. One family member is pC221 and the initiation of its replication, occurs when RepD, a homodimeric, topoisomerase I-like enzyme^2^, binds at the double-stranded origin of replication, *oriD*, which contains three inverted complementary repeat sequences (ICRs), termed ICRI, ICRII and ICRIII. It binds tightly and specifically to ICRIII (*K*_d_ = 10^−8^ M^3^) and rapidly nicks^4^ one DNA strand within ICRII^5^ and, as a result, DNA supercoiling is relaxed due to free rotation around single bonds in the backbone structure of the single-stranded DNA. The exposed single-stranded region, allows a helicase and DNA polymerase to bind and together unwind and copy the leading strand^6,7^ (Fig. 1). During nicking, the active site Tyr188 of RepD forms a covalent bond with the 5′-end of the DNA and it remains bound until the termination of replication^8^. In the final stage, DNA is supercoiled again by gyrase activity.

*In vitro* experiments have shown that the RepD nicking reaction is very slowly reversible (rate constant 0.004 s^−1^) and religation gives rise to a closed, relaxed, plasmid^4^. At equilibrium the nicked and religated plasmid products are present in roughly equal amounts and the equilibrium constant is close to unity. This means the rate at which RepD nicks a relaxed plasmid is similar to the rate of religation and much slower than the nicking rate measured on supercoiled plasmid. Therefore DNA topology, in some way, has a profound effect on RepD nicking activity.

More generally, we know that supercoiling-induced changes of DNA topology have significant and diverse effects on protein-DNA interactions. Build-up of positive supercoiling ahead of DNA replication or transcription machinery must be relieved in order to prevent such activities from stalling^9^ and negative twist tends to open the DNA duplex, which accelerates processes requiring access to the DNA bases^10–13^. Another consequence of negative twist is to stabilize alternative DNA structures, such as cruciforms and hairpins, which can form at regions where there are inverted complementary repeats, or ICRs, in the DNA^14^.

It has long been suspected that secondary structure formation at the ICRs of *oriD* might be important for site recognition and nicking by RepD, in particular, hairpin formation at ICRII would expose the nick site on a single-strand loop. A recent structural study^15^ provides strong support for this idea as the reported 3-dimensional structure of RepD indicates *oriD* would need to be bent in order to accommodate simultaneous binding at ICRIII and positioning of ICRII close to the active site Tyr188. Recently, the activity of an homologous protein, RepC, was shown to be supercoiling-sensitive^16^. However, direct evidence explaining the supercoiling-sensitivity is still missing.

**Figure 2:**
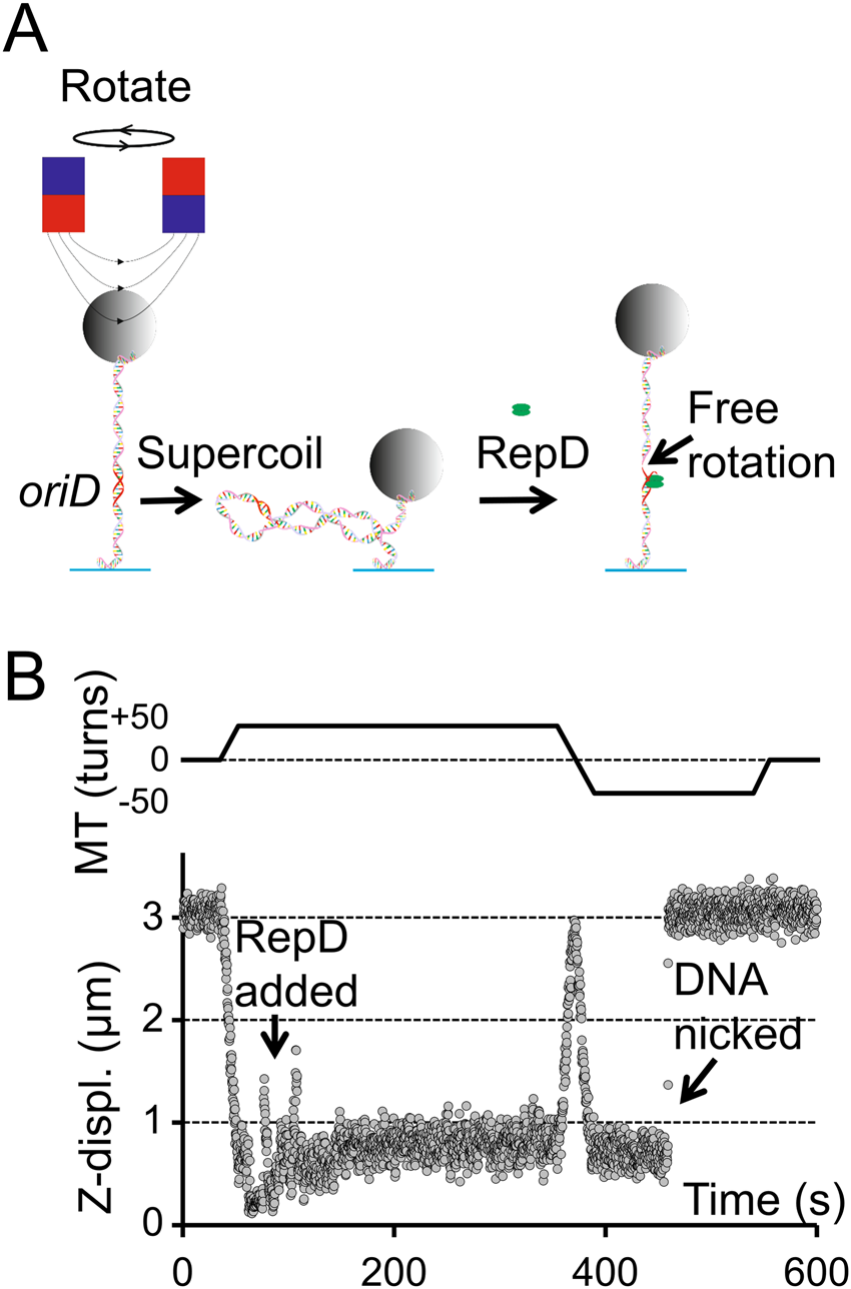
Magnetic tweezers RepD nicking assay. (**A**) A 1 μm diameter, paramagnetic bead is attached by multiple biotin-streptavidin linkages to one end of a dsDNA (4 kb or 10 kb) molecule that has the initiation site, *oriD*, within its central region. Multiple digoxigenin-anti-digoxigenin linkages attach the other end to a glass microscope coverslip. Application of a magnetic field, generated by a pair of permanent magnets, causes the bead to rise from the microscope coverslip surface, extending the DNA molecule. Rotation of the magnetic field causes bead rotation and DNA supercoiling. At low force, the dsDNA first becomes twisted and then undergoes plectoneme formation (DNA writhe) resulting in reduction in DNA end-to-end length and bead motion towards the coverslip surface. When RepD nicks the DNA, supercoiling is relaxed and the bead moves rapidly upwards, towards its extended length. (**B**) The upper trace shows rotation of the magnetic tweezers (MT) and the lower trace shows bead displacement relative to its rest height plotted as a function of time. At the start of the experiment (t = 50 s), the DNA is supercoiled positively by 50 turns, increasing its super-helical density, σ, by (50 × (10.5/10 kb)) = +5% which causes plectoneme formation and bead height to reduce. RepD (1 nM) is then flowed into the experimental chamber (down-arrow, t = 80 s) and after 250 s the DNA remains intact and supercoiled. The magnetic tweezers are then rotated counter-clockwise by 100 turns (at t = 350 s) so that the DNA is then negatively supercoiled by −50 turns (σ = −5%). After a stochastic delay (here, ~50 s) the DNA is nicked (diagonal arrow) and the bead moves rapidly back to rest height. After a further 100 s (at t ~ 525 s), rotation of the magnetic tweezers causes the magnetic bead to rotate but no longer causes the DNA to become supercoiled. The applied force due to the magnetic field was 0.4 pN and temperature was 23 °C.

In the current work, we have investigated the effect of supercoiling and associated structural changes on the activity of RepD by applying known amounts of DNA supercoiling and monitoring RepD nicking at the single molecule level using magnetic tweezers^17^ (Fig. 2A). We found that RepD nicking activity is controlled by DNA supercoiling and is blocked by positive supercoiling and activated by small amounts of negative supercoiling. Our results also show that regions of DNA within *oriD* form secondary structures when negative supercoiling is applied and the structures are stabilized by RepD binding. We used bulk rapid-reaction methods (quenched-flow) to investigate the effect of *oriD* mutations on RepD nicking. We conclude that the ICRII within *oriD* serves both as a unique start site for replication and because of its propensity to form secondary structures, as a mechanical checkpoint that ensures replication can only commence when the plasmid DNA is supercoiled, an indication that DNA is not damaged or a previous cycle of replication has fully finished.

## Results

### Positive supercoiling blocks RepD activity

In order to determine the effect of DNA supercoiling on the kinetics of the RepD nicking reaction we constructed 10-kb and 4-kb, double-stranded DNA (dsDNA) linear templates with centrally located *oriD* sequence, flanked on either side by lengths of dsDNA “handles” that allowed specific attachment at one end to the surface of a microscope coverslip and at the other to a super paramagnetic bead (~1 μm diameter)^18^. Experimental conditions were optimized such that 5–15 beads, each tethered by a single DNA molecule via both DNA strands could be observed and recorded simultaneously by bright-field video microscopy. When a polarized magnetic field was applied, the easy axis of the paramagnetic beads became aligned with the magnetic dipole so that when the field was rotated the beads also turned and the attached DNA became supercoiled. At low stretching force (<0.5 pN), supercoiling was initially taken up by DNA twist but after a few turns the molecule buckled and underwent writhe with consequent plectoneme formation causing shortening of ~70 nm for each additional turn and this behavior was roughly symmetrical for positive and negative supercoiling (Fig. S1). When RepD was added to the experimental chamber and DNA was negatively supercoiled, the nicking reaction caused sudden loss of supercoiling (Fig. 2B). The DNA relaxed back to its rest length and further magnetic field rotation no longer caused it to supercoil. The nicking reaction could therefore be monitored directly from changes in DNA length (i.e. z-displacement of the attached paramagnetic bead) or because the DNA molecule was no longer able to be supercoiled.

The striking initial observation was that when the 10 kb-DNA template was positively supercoiled by +50 turns (superhelical density, σ = +5%), it was completely resistant to RepD nicking even after several hours incubation. Conversely, when the DNA was subjected to physiological levels of negative supercoiling (−50 turns, σ = −5%) it was nicked within a few seconds of RepD addition (Table 1).

**Table 1:** Effect of DNA supercoiling on RepD nicking activity.

Supercoiling	Force = 0.4 pN	Force = 0.8 pN
+50 turns (σ = +5%)	0% (n = 10)	0% (n = 36)
−50 turns (σ = −5%)	100% (n = 10)	100% (n = 21)

10-kb dsDNA templates with *oriD* were held using the magnetic tweezers at fixed amounts of either positive or negative supercoiling at two different forces (0.4 and 0.8 pN) while incubated with RepD (100 nM). The proportion of nicked DNA molecules was then counted by noting the change in DNA length after 10 minutes’ incubation.

**Figure 3:**
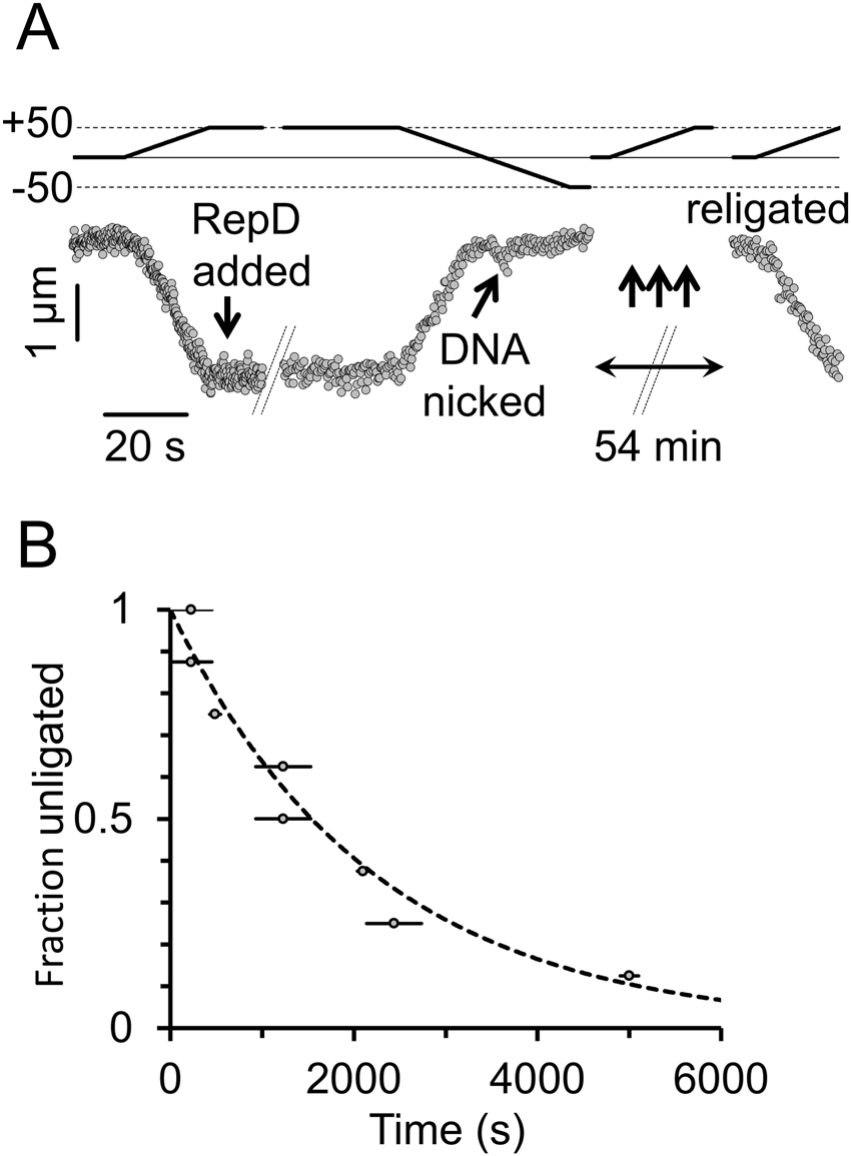
DNA nick-religation activity by RepD. (**A**) Upper trace (solid line) shows rotational position of the magnetic tweezers and the lower trace (filled circles) shows the bead height as a function of time. The 10-kb dsDNA was first positively supercoiled (by +50 turns) and then RepD (100 nM) was added (down-arrow). The magnetic field was then rotated (by −100 turns) driving the bead and associated DNA molecule toward 50 turns of negative supercoiling. In this example, the DNA molecule was nicked (diagonal arrow) almost as soon as it started to enter the regime of negative supercoiling. Because the DNA had been nicked it could no longer undergo the characteristic length changes associated with supercoiling. Repeated cycles of 50 positive turns of field rotation were then applied to test if the DNA molecule was “supercoilable”. Here, after 54 minutes, the DNA spontaneously religated, and could again be supercoiled. (**B**) Cumulative frequency plot showing the number of nicked DNA molecules remaining as a function of time (n = 8). The mean time for religation to occur was 1,420 seconds (~24 minutes), giving a rate constant (dotted line) of 5 × 10^−4^ s^−1^. The horizontal lines indicate timing uncertainty due to gaps between applications of the +50 turn test protocol (see **(A)** above). Experiments were at F = 0.4 pN and 23 °C.

We tested for nick site religation by repeatedly rotating the magnetic field through 50 positive turns at five minute intervals over a period of up to 2 hours and scored the number of DNA molecules for which torsional continuity had been restored so that the DNA molecule could again be supercoiled (Fig. 3A). We found the half-time for nick religation was ~24 minutes (Fig. 3B), which is about seven times slower than measured in bulk assays^4^ (previously performed at 30 °C and the current experiments which were at 23 °C). The discrepancy may be explained by the temperature difference and also the fact that we hold the DNA under a small amount of tension (0.5 pN) in the magnetic tweezers experiments, potentially making it less favorable for the DNA 3′ end to bind RepD and undergo the phophodiester exchange reaction. Saturating concentrations of RepD (100 nM) were used, and several flow-cell volumes were exchanged during addition, so it is unlikely that depletion of RepD by non-specific binding to surfaces would have a significant affect, since its affinity for DNA is ~6 nM^3^.

### RepD is activated by small amounts of negative supercoiling

**Figure 4:**
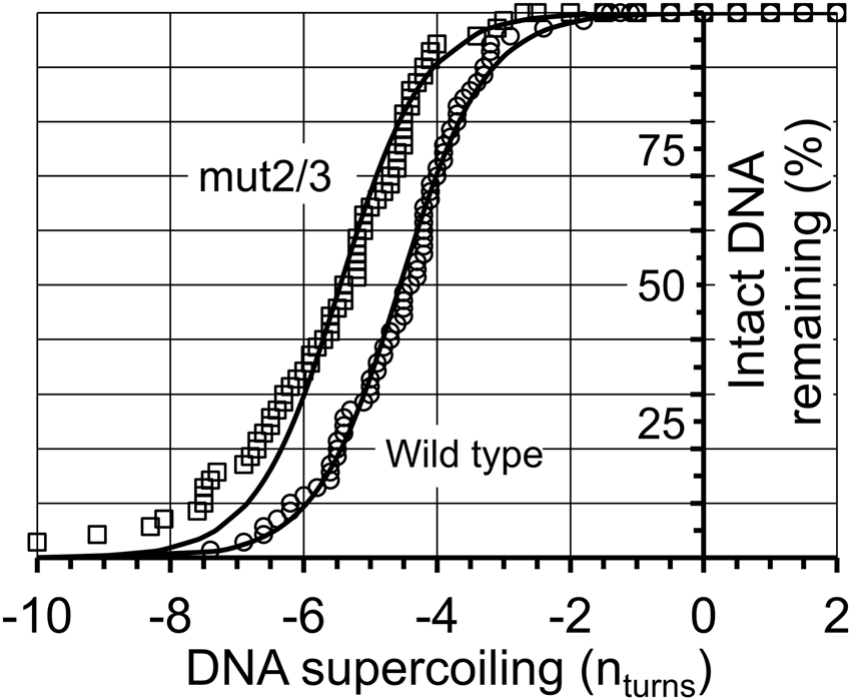
dsDNA is nicked by RepD only when it is subjected to negative supercoiling. Beads that were tethered to the surface by a single, 4-kb, dsDNA molecule were identified by the characteristic change in DNA length upon supercoiling (n_obs_ = ~70). The DNA molecules were then positively supercoiled by +20 turns before 100 nM RepD was added to the experimental flow-cell. All DNA molecules remained intact until they were subjected to small levels of negative supercoiling. After −4.5 ± 0.1 turns (±SEM) of negative supercoiling (σ = −1.2%), 50% of the DNA molecules with the wild type *oriD* sequence (circles) were nicked by RepD. The *oriD* mutant mut2/3 (squares), had the 50% nicking threshold at −5.6 ± 0.2 turns. Experiments were at F = 0.4 pN and 23 °C. The exact level of supercoiling for each DNA molecule was corrected for its initial starting offset due to thermal motion (see main text for details). At low levels of supercoiling, the elastic energy (Δwork) due to changes in DNA torque and secondary structure formation approximates to the function: $\frac{C}{2{{\rm{l}}}_{{\rm{O}}}}[{(2{\rm{\pi }}{\rm{n}}+2{\rm{\pi }}h)}^{2}-{(2{\rm{\pi }}{\rm{n}})}^{2}]$ where C is DNA torsional stiffness (240 pN.nm^2^.rad^−1^ per unit length^27^), l_o_ is the DNA length (here, 4000 bp * 0.34 nm/bp = 1360 nm), n, the number of supercoiling turns and h, the number of helical turns transferred from dsDNA backbone into hairpin structure. E_loop_ is the enthalpic energy cost of unstacking and unpairing bases in the DNA loop regions (see main text). The least-squares, fitted-lines are to the relationship: $y={(1+ex{p}^{(\frac{-{E}_{loop}-{\rm{\Delta }}work}{{{\rm{k}}}_{{\rm{b}}}{\rm{T}}})})}^{-1}$ Fitting parameters were: wild type *oriD*: h = 0.91 turns and E_loop_ = 31 pN.nm; and mut2/3: h = 0.71 turns and E_loop_ = 29 pN.nm. The leftward shift of the mut2/3 *oriD* data compared to wild type is equivalent to an additional torsional energy requirement of ~34 pN.nm.

To determine the threshold of supercoiling required for RepD nicking to occur, we developed an experimental protocol that allowed the nicking reaction to be monitored at very low levels of DNA supercoiling, where bead z-displacement is minimal: First, all of the DNA molecules within the field of view were positively and then negatively supercoiled by rotating the magnetic field by +50 through to −50 turns. This allowed the thermally-driven starting variation in linking number for each DNA molecule to be accurately determined (Fig. S1). The DNA molecules were then positively supercoiled by applying +20 turns of the magnetic field and RepD (100 nM) was added to the experimental chamber. The supercoiled DNA molecules were then successively unwound by rotating the magnetic field to test points of +6, +4, +2, 0, −2, −4, etc. turns, relative to the nominal zero starting value where, on average, the DNA molecules were in their relaxed B-state. This therefore gave a spread of DNA supercoiling values at each test point due to the dispersion in starting offset values for each molecule across the field of view. The DNA molecules were held at each test point for 2 minutes and then rewound by +20 turns so that the number of intact (un-nicked) molecules could be counted by checking which of the DNA molecules underwent plectoneme formation and characteristic reduction in bead-height (z-displacement). When wild-type, *oriD*-containing, DNA molecules were held either in a positively supercoiled or relaxed state they were completely resistant to nicking by RepD (as found above). However, when small amounts of negative-supercoiling were applied, the RepD nicking reaction was activated and 50% of the DNA molecules were nicked with 4.5 turns of negative supercoiling (σ ~ −1.18%) (Fig. 4).

The shape and position of the curve, relating RepD nicking to DNA supercoiling, gives information about the energetics of the system. If we assume that negative supercoiling, which tends to unwind the dsDNA helix, causes local melting^10^ then regions of dsDNA duplex containing ICRs may separate, allowing complementary base-pairing to occur within the same DNA strand. This would give rise to a hairpin structure with a net enthalpic cost, E_loop_, due to creation of non-base-paired loop regions. When a hairpin stem forms at inverse complementary repeat regions, each helical turn (~10.5 bp), transferred from dsDNA backbone into hairpin stem, results in addition of one turn of positive supercoiling to the main dsDNA backbone^19^. Depending on the supercoiled state of the DNA this will either reduce negative twist or increase positive twist. The data are described well by this relationship, allowing estimates of the hairpin stem length, “*h*” and enthalpic cost, “E_loop_” (see Fig. 4 legend) to be obtained by least-squares curve fitting.

To explore further the importance of secondary structure formation at *oriD*, we compared results from wild-type *oriD* (above) to a mutant, in which both ICRII and ICRIII had been disrupted by scrambling the inverted complementary repeat regions while retaining the RepD consensus binding and nicking sites^3,4,20^ (see Table 2). We found that 50% activation of RepD nicking activity required the mutant, mut2/3, DNA molecules to be negatively supercoiled to a greater extent; 5.5 turns (σ = −1.5%). The leftward shift implies more torsional energy is required to activate the nicking reaction on the mut2/3 *oriD*

**Table 2:**
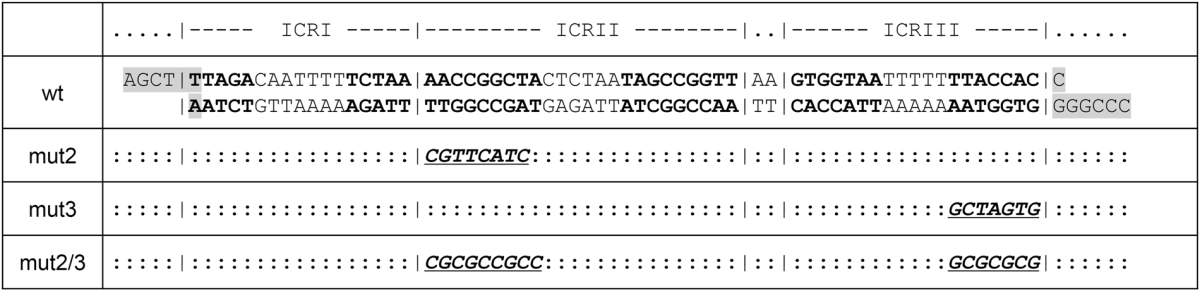
Mutant *oriD* inverted complementary repeat (ICR) sequences.

Mutant *oriD* oligoduplexes were synthesized and then ligated into the wild type (wt) pCer*OriD* plasmid to yield three *oriD* variants mut2, mut3 and mut2/3. The following symbols are used; “:” identical to wt; “|” delimits the three ICRs, bold indicates hairpin stems (see Fig. S3C); shaded regions are the restriction enzyme sites (HindIII and AvaI) used to resect the wild type pCer*OriD* plasmid so the synthesized oligoduplexes could be inserted and religated.

.

### Negative supercoiling causes DNA length fluctuations

**Figure 5:**
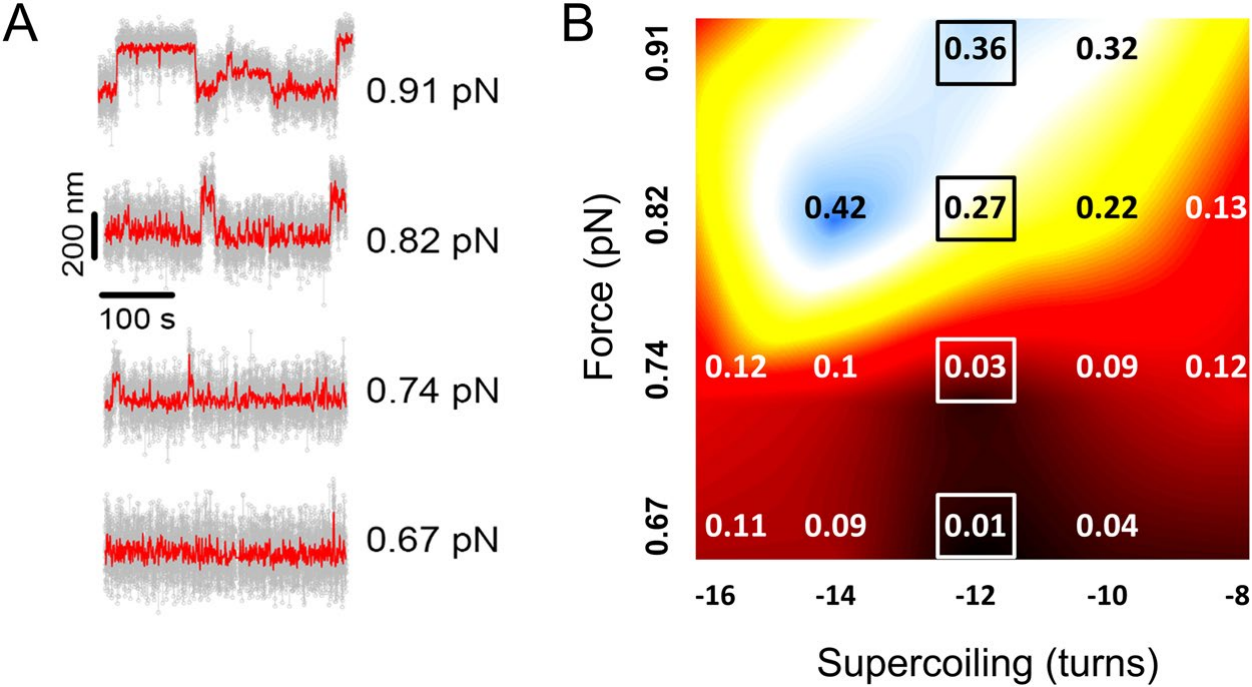
Negatively-supercoiled DNA held at low force undergoes length fluctuations. Large amplitude length fluctuations occurred when 4 kbp, *oriD*-containing, DNA templates were negatively supercoiled (by −8 to −16 turns) and held at 0.67 to 0.91 pN load. (**A**) Example traces showing DNA length fluctuations at −12 turns of supercoiling and a range forces. Grey points are raw data and red lines (overlaid) are the running average of 25 points. (**B**) Data point values show the fraction of time the DNA molecule spent at the extended length at different forces and supercoiling levels. Pseudo-color coded background (from black to blue) is an interpolated heat-map to illustrate the fraction of time in the alternative state at each load and supercoiling level. The data points were obtained by histogramming z-displacement data (from 20–25 molecules, recorded for >300 seconds for each condition) so that the proportion of time spent in the extended state could then be determined by fitting to a dual Gaussian function (see Fig. S3 for details). The boxed values are from the same conditions as the raw data records in (**A**). The SEM of each point is around ±0.05. Experiments were performed at 23 °C, K100 + buffer.

To explore the structural stability of the ICRs at *oriD* a 4-kb DNA template was used in order to increase the signal-to-noise ratio of our measurements and also to eliminate other, non-*oriD*, ICRs. When the DNA molecules were held at 0.67 to 0.91 pN load and subjected to negative supercoiling (from −8 to −16 turns, i.e. σ from −2% to −4.2%) in the absence of RepD, they exhibited large amplitude length fluctuations that occurred on a timescale of a few seconds (Fig. 5). With increased force and more negative supercoiling, DNA spent more time in these extended-length alternative states. When the experiment was conducted at higher temperature (26 °C) and using a low-salt buffer (containing no added KCl or MgCl_2_), fluctuations occurred at lower force 0.4 pN (Fig. S2). Notably, the fluctuations did not occur when the DNA was held at the same degree of positive supercoiling (σ ~ +2.5%).

This phenomenon has been observed before^19^ and can be explained by formation and collapse of secondary structure at the ICRs with concomitant loss and gain of DNA writhe. Each turn of the B-form DNA backbone helix (10.5 bases) converted into the hairpin structure relieves one turn of negative supercoiling (or generates an additional turn of positive supercoiling). When the DNA is negatively supercoiled and in writhe, conversion of B-form DNA to a hairpin structure results in a large change in end-to-end length, because each turn of writhe that is lost causes a ~70 nm vertical bead movement at 0.4 pN force.

### *oriD*-dependent length fluctuations are stabilized by RepD binding

**Figure 6:**
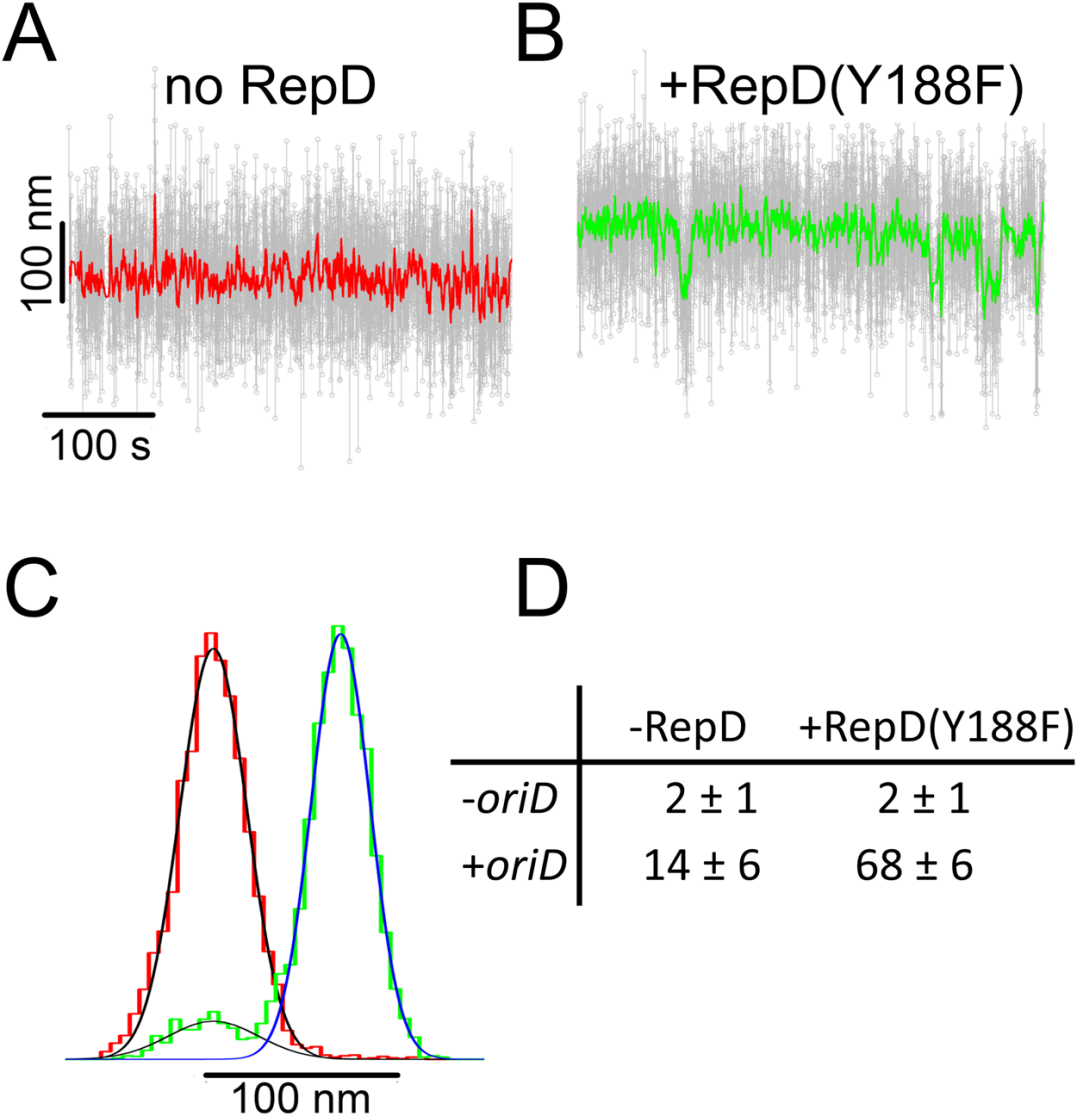
RepDY188F stabilizes hairpin formation at *oriD*. (**A**) A 4-kb *oriD* DNA molecule, held at the conditions when DNA fluctuations are still sparse (−10 turns 0.67 pN), shows occasional elongation events (i.e. upward movements). Raw data is shown as grey circles; colored lines are 25-point running averages. (**B**) After addition of RepDY188F (60 nM) the DNA molecule is biased towards the elongated state and the mean length is ~80 nm longer (equivalent to loss of about 1.5 turns of writhe) and now occasionally exhibits downward movements. (**C**) The averaged, z-displacement data for data shown in records “A” (red) and “B” (green) are plotted as histograms with dual Gaussian fits (continuous lines, see Fig. S3 for further details). The area under the curves gives the fraction of time spent in each state. (**D**) The fraction of time (%) spent in the elongated (hairpin) state is tabulated for different conditions, +/−RepDY188F (60 nM) in the bathing solution and +/− *oriD* site in the DNA template (n = 25 DNA molecules for each condition). All experiments were at −10 turns negative supercoiling (i.e. σ = −2.6%), F = 0.67 pN and 23 °C.

**Figure 7:**
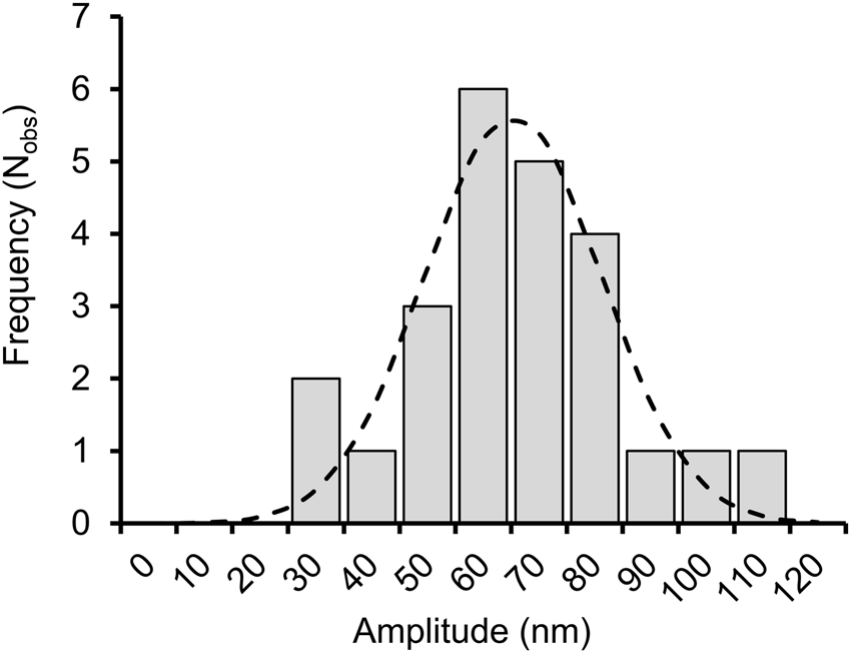
Fluctuations in DNA length with central *oriD* sequence after addition of RepDY188F. The amplitude of DNA length fluctuations due to alternation between B-state DNA and hairpin secondary structure formation was determined from the distance between the means used to fit the twin Gaussian distributions to the histogrammed bead height data. The average amplitude of the length fluctuations is 71.5 nm and standard deviation is 22.4 nm (least-squares fit to the data, dashed line). Given that 1 turn of writhe produces a 60 nm length change at 0.67 pN; the number of base pairs involved in the structural rearrangement = (71.5/60) *10.5 = 12.5 ± 3.4 bp. Experiments were conducted at 0.67 pN, K100 + buffer and 23 °C.

We next used a non-nicking variant, RepDY188F, which retains sequence-specific binding to *oriD*^8^, to explore whether RepD binding at *oriD* might affect the stability of alternative DNA structures. The supercoiling and force conditions were chosen so alternative structure formation just starts appearing and non-specific (less-stable) structures are not yet stabilized. When the 4-kb dsDNA template was held at 0.67 pN force and negatively supercoiled by −10 turns (σ ~ −2.6%) it exhibited length fluctuations, spending ~14% of its time in the longer state (Fig. 6). After addition of RepDY188F the proportion of time in the elongated state increased to 68%. Under these conditions dwell times were sufficiently long to allow the fluctuation amplitudes to be measured directly from the raw data and histogrammed (Fig. 7). The mean amplitude, 71.5 nm, is equivalent to 1.2 turns of writhe consistent with ~12 base pairs interconverting between conventional backbone dsDNA helix and an alternative hairpin structure which implies that at least one of the ICRs at *oriD* forms a hairpin structure as RepD binds.

To verify that DNA length fluctuations were due to *oriD*-specific binding of RepDY188F, a DNA template with no *oriD* sequence was tested and no length fluctuations were observed either before or after addition of RepDY188F, supporting the idea that RepD binding stabilizes DNA structural rearrangements that occur specifically at *oriD*. The ~10-fold change in equilibrium constant, between B-state and alternative structure in *oriD*, brought about by RepDY188F addition implies the binding energy is around −6.3 kJ.mol^−1^ (10.7 pN.nm per molecule).

When the experiment was repeated at lower force (0.4 pN) the noise level was higher, therefore accurately measuring the time spent in both states was challenging (Fig. S3). However, a similar trend was observed and after addition of RepDY188F the DNA-length histograms were skewed towards the extended lengths.

### RepD nicking is ~10-fold slower when ICRII sequence is scrambled

**Figure 8:**
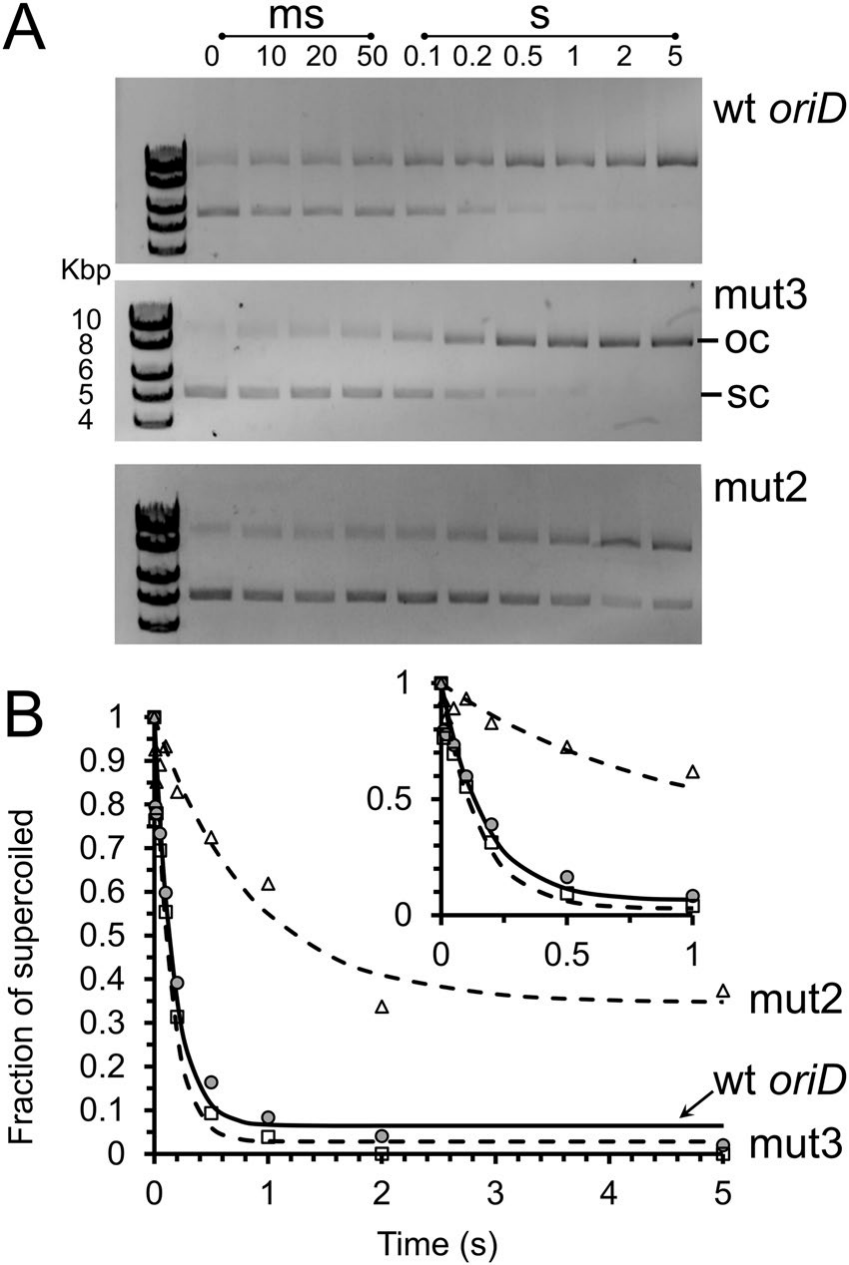
Kinetics of supercoiled plasmid nicking. (**A**) Intact natively-supercoiled (10 kbp) plasmid was rapidly mixed with RepD in K100 buffer at 23 °C. The reaction was quenched by addition of excess EDTA at the time points noted above the gel lanes. The reaction products were electrophoresed on 1% agarose gels in the presence of ethidium bromide. Lower band was the fast-migrating intact, supercoiled plasmid (sc), the upper band, is the slow-migrating nicked, open circular form (oc). (**B**) Quantitation of RepD nicking by gel densitometry gave the proportion nicked (normalized as oc/(oc + sc)) as a function of time. The least-squares fitted lines are to a single exponential function, giving rate constants, 6 s^−1^, 7 s^−1^ and 1.2 s^−1^ (wt *oriD*, filled-circles solid-line; mut2 open-triangles, dotted-line, and mut3, open-squares, dotted-line). The inset plot shows data on a faster timescale. RepD nicking of wt *oriD* and mut3 was complete whereas mut2 had an amplitude of 60%. Experiments were conducted at 23 °C.

In order to understand how the structure of *oriD* affects RepD activity we performed a series of rapid-mixing quenched-flow experiments to measure the RepD nicking rate using intact, natively-supercoiled, plasmids. The plasmids contained either wild-type *oriD* or variants in which ICRII (mut2) or ICRIII (mut3) were disrupted (see Table 2). The nicking reaction was initiated by rapidly mixing intact, supercoiled, plasmid with RepD using the same buffer (K100) used for our magnetic tweezers experiments. The reaction was then stopped (i.e. quenched) at various time points by addition of excess EDTA. It took ~150 ms for RepD to nick 50% of the wild-type *oriD* plasmid and the mut3 mutant (Fig. 8). However, nicking of the ICRII disrupted mutant (mut2) was ~10-fold slower, taking >1.5 s for 50% nicking, and after 5 seconds only 60% of the plasmids had been nicked. Thus the main effect of the mutant *oriD* (mut2/3) used in the magnetic tweezers measurements is likely to be due to disruption of ICRII; the ICRIII mutation may affect tightness of binding, but at the RepD concentrations used in these experiments, binding is likely to be quantitative.

## Discussion

The work presented here defines the relationship between supercoiling and effectiveness of RepD nicking at *oriD*. Because DNA nicking by RepD is an absolute requirement for initiation of rolling-circle plasmid replication, this relationship provides important information on a critical control point. We conclude that negative supercoiling causes ICRs at *oriD* to form secondary structure and that RepD binding shifts the equilibrium in favor of such structures. Although our data suggest that only one ICR is stabilized upon RepD binding, more ICRs can fold upon higher level of negative supercoiling. Because the ICRs are mechanically linked in series with one another, when one ICR motif converts into a stem-loop structure it relieves supercoiling, stabilizing the remaining ICRs. Although activation of RepD nicking has a relatively low requirement for supercoiling (just 4 turns applied to a 4kbp DNA molecule, σ = 1.2%) in fact the amount of energy needed to reach that degree of supercoiling is still 10-fold greater than thermal energy. Thermal fluctuations would rarely reach such an extreme value and the energy barrier would give >1000-fold slower reaction rate. Although the equilibrium constant for nicking and religation of relaxed plasmid is close to unity, nicking appears inefficient because of the slow forward rate (~0.004 s^−1^). Negative supercoiling acts as a strong control/regulatory factor because it stimulates the rate of RepD nicking by >1000-fold. We show this both in our quenched-flow (using natively supercoiled plasmid) and magnetic tweezers experiments (where we precisely control supercoiling).

The dependence of the extent of nicking on the amount of supercoiling is well-fitted by a simple (non-cooperative) model of hairpin formation (Fig. 4) that is thermodynamically favored when DNA is negatively-supercoiled. Our finding strongly suggests that hairpin structure formation is a prerequisite for nicking. The idea that RepD can only nick effectively when these secondary structural motifs have formed is supported by recent structural studies of RepD (pdb:4CWE^15^). In fully double-stranded *oriD*, the linear distance between the ICRIII binding site and the nucleotide base in ICRII that is nicked by the RepD catalytic residue is ~8 nm. This is much greater than the separation of the corresponding amino acid residues on the proposed RepD structure (~4.5 nm). Furthermore, the near orthogonal geometry of the corresponding regions in the RepD structure also argues that there should be a matching 90° bend between ICRII and ICRIII (as proposed in reference^15^). Together this suggests there is a structural rearrangement at *oriD* allowing RepD to bind at ICRIII while contacting the nick site within ICRII.

We found that at critical levels of tension and supercoiling the DNA molecules showed large amplitude length fluctuations (measured by changes in bead displacement). Fluctuations occurred when DNA was negatively but not positively supercoiled and only when the ICRs at *oriD* were present. The finding is most easily explained by transient secondary (hairpin) structure formation^19^ at the inverted complementary repeat regions of *oriD* that gives rise to stochastic loss and gain of DNA writhe. The increased dwell-time of the extended state in the presence of the non-nicking RepD mutant protein (RepDY188F) can be explained by hairpin structure stabilization as the protein binds (Figs 5, 6 & S3). The change in equilibrium constant gives an estimate of the binding energy of RepD, −6 kJ.mol^−1^, which would likely be insufficient to drive hairpin formation on relaxed DNA.

When DNA is negatively supercoiled, elastic energy is stored in both twist and writhe and this stored energy is dissipated by hairpin structure formation. Conversely energy stored when DNA is positively supercoiled acts against hairpin formation. At low levels of supercoiling the DNA becomes twisted and energy associated with its torsional distortion has a quadratic dependence on the number of supercoiling turns, “n”; given by: $\frac{1}{2}\kappa {(2\pi n)}^{2}$, where κ i.s the torsional stiffness (see Fig. 3 legend). At a critical torque force, DNA buckles (called the buckling transition) and then undergoes writhe whereby the DNA backbone wraps around itself to produce plectonemes. In our magnetic tweezers experiments, DNA tension is held constant and energy stored as writhe increases linearly with the number of additional turns. At low force, each additional turn of writhe results in a change in DNA end-to-end length of ~70 nm (Fig. S1C) which at 0.4 pN load gives an energy change of −28 pN.nm (16.5 kJ.mol^−1^). When a hairpin structure forms, entropic energy stored as both twist and writhe is lost from the DNA backbone and this counteracts (or pays) the enthalpic cost of forming unstacked and unpaired bases in the hairpin loop regions. We found RepD nicking activity had a characteristic dependence on supercoiling and was activated by 4.5 turns of negative supercoiling applied to a 4-kb length of DNA (σ ~ −1.3%), which, coincidentally, is close to the buckling transition. Using hairpin loop energy cost values from Table 4 in ref.^21^ the free energy change associated with hairpin loop formation at ICRI, II and III (with loop sizes 7, 6, 5 bases resp.), is: +17.6, +16.8, +13.9 kJ.mol^−1^; which in units of mechanical work per dsDNA molecule gives +59, +56, +46 pN.nm for each loop pair (resp.). If we consider, for example, the enthalpic cost of forming the two loop regions at the ICRII cruciform, E_loop_ = +56 pN.nm, compared to the entropic energy available from loss of negative supercoiling due to the change in linking number when the 24 bp making up ICRII are transferred from the linear DNA backbone to the hairpin stem structure (24/10.5 = 2.29 turns); we obtain Δwork ~ −60 pN.nm (at 0.4 pN load, and z-displacement of ~70 nm per turn of writhe).

Binding of a homologous initiator, RepC, to *ori*C was found to protrude single-stranded DNA regions consistent with hairpin formation^22^. Also, RepC binding to short *ori*C DNA templates caused the DNA to bend about *ori*C^23^. Recent work using magnetic tweezers showed RepC supercoiling-sensitivity^16^. Our results are consistent with these studies and extend their findings by directly showing the extrusion of cruciform structures in *oriD*. Our data support the formation of ICRII upon RepD binding. These results strongly suggest the important role of cruciform extrusion in controlling the rolling circle replication. We note that other regulation points in initiation of replication may include control of RepD synthesis^24^ and that RepD is inactivated after the replication cycle is complete^25^.

The energy of supercoiling can drive the initiation process to completion prior to the NTP-driven processes of DNA unwinding and DNA synthesis. RepD can only nick when the DNA is negatively supercoiled, hence replication can commence only when the plasmid is topologically intact. Furthermore, if the plasmid retains rotational freedom behind the replication fork it would not be negatively supercoiled until the leading strand had been fully replicated and single strand nick resealed by the termination reaction. This means replication cannot be erroneously initiated at the newly synthesized *oriD* site until the previous replication cycle is complete and the intact daughter plasmid has become negatively supercoiled. This is important to ensure a faithful copy of the original plasmid is created.

## Materials and Methods

All reagents were sourced from Sigma-Aldrich Co. (Gillingham, Dorset, UK) unless stated otherwise; TFS (ThermoFisher Scientific, Hemel Hempstead, Herts, UK), NEB (New England Biolabs, Hitchin, Herts, UK), Agilent (Agilent Technologies, Stockport, Cheshire, UK).

### DNA constructs

The 10-kb and 4-kb dsDNA templates were each constructed from 3 separate pieces of dsDNA that were made by PCR: A 500-bp digoxigenin-labelled “handle”; a 500-bp biotin-labelled “handle” and a central fragment of either 9.5-kb or 3.6-kb which had *oriD* located approximately in the middle. All three pieces of DNA were created from a synthetic, 10-kb plasmid based on pCer*OriD*^3^. The central fragments of DNA, used for the magnetic tweezers experiments, were generated from pCer*OriD* using suitable primers and PfuUltra II Fusion HS DNA polymerase (Agilent) according to the manufacturer’s instructions. The products were purified and digested with FastDigest AscI and FastDigest ApaI simultaneously. The 4-kb DNA fragment, without *oriD*, was prepared from a pCer*OriD* 10-kb plasmid which previously had *oriD* deleted using the QuikChange technique (Agilent) and suitable primers. In order to create the differentially end-labelled dsDNA templates the two differentially-labelled DNA handles (below) were mixed with the chosen central fragment (10-kb or 4-kb) at a molar ratio of 20:20:1 and ligated using T4 DNA ligase (NEB), for 2.5 h at room temperature in T4 ligase buffer. The final product was gel-purified and stored in small aliquots at −80 °C in 1 mM EDTA, 10 mM Tris.HCl pH 8.0.

### Differentially-labeled DNA handles

In order to create the digoxigenin- and biotin-labelled dsDNA handles a ~1000-bp piece of DNA was generated by PCR, using 0.5 ng/µl pCer*OriD* DNA, 1.25 u/µl recombinant Taq Polymerase (TFS), Taq polymerase buffer with 2 mM MgCl_2_, 0.2 mM dNTP (each) and 1 µM of suitable primers and in the presence of either 0.02 mM digoxigenin-11-dUTP or biotin-16-dUTP. The PCR product was purified with a PCR purification kit (QIAGEN, Manchester, UK), and treated with a FastDigest (TFS) restriction enzyme; using ApaI for the digoxigenin-labeled handle and AscI for the biotin-labeled handle, according to the manufacturer’s instructions. After digestion the enzyme was inactivated at 60 °C for 20 min and DNA was purified again. By using this protocol 1000-bp and 500-bp handles were produced for 4-kb and 10-kb DNA constructs respectively.

### Mutant *oriD* plasmids

10-kb pCer*OriD* with wild type (wt) *oriD* was mutated 10-kb pCer*OriD* with wt *oriD* was mutated by excising the *oriD* sequence (AvaI and HindIII (NEB)) and ligating the required, 5′ phosphorylated, mutant *oriD* oligoduplexes (Integrated DNA Technologies, Leuven, Belgium), with T4 ligase (NEB). This gave three variants; mut2 with disrupted ICRII, mut3 with disrupted ICRIII, and mut2/3 with both ICRII and ICRIII regions disrupted. The inverted complementary repeat sections that form stem structures were mutated but the central loop regions were left unchanged (see Table 2).

### Protein production

We used RepD cloned into pET11a following the method described by Thomas *et al*.^5^. Briefly, the pET11a plasmid with cloned RepD gene was expressed in B834 (λDE3) pLysS cells at 30 °C. Cell pellets were sonicated and protein was salted-out using saturated (NH_4_)_2_SO_4_. The product was cleaned with HiTrap Q FF column and bound on to HiPrep heparin column. Protein was eluted with a KCl gradient (50 mM Tris.HCl pH 7.5, 1 mM EDTA, 10% (v/v) ethanediol, and 200-500 mM KCl), filtered through a 0.45 μm filter and concentrated using 10,000 MWCO Vivaspin concentrator Millipore (Millipore, Watford, Herts, UK). The final RepD product was quantified using an extinction coefficient of 119,514 M-1.cm-1 at 280 nm for the dimer. The protein solution was stored frozen at −20 °C. To produce the non-nicking, RepDY188F mutant, we used the QuikChange technique (Agilent) using 58-base primers having the desired, centrally located tyrosine to phenylalanine point mutation (i.e…atttAtaat.. to..atttTtaat..) on forward and reverse primers.

### Quenched-flow

RepD (15 μl of 70 nMolar = 10 pmoles) was mixed with an equal volume of 10-kb pCer*OriD* plasmid (20 ng/µl = 0.045 pmol of *oriD* sites) in a rapid-mix quenched-flow apparatus (QFM-4000, Bio-logic Science Instruments, Seyssinet-Pariset, France) using a 3.5 μl delay-line. Both reactants were in K100 buffer (100 mM Tris.HCl, pH 7.5, 100 mM KCl, 10 mM MgCl_2_, 1 mM EDTA). The reaction was stopped at various times by controlled addition of EDTA (total volume, 15 μl of 150 mM = 50 mM final concentration) at 23 °C. Products were collected and then separated by DNA electrophoresis on 0.8% agarose gel in the presence of 1 μg/ml ethidium bromide in TAE buffer (40 mM Tris pH 8.0, 20 mM acetic acid, and 1 mM EDTA). After running the gel, quantitative estimation of closed and open plasmid products was achieved by imaging by Amersham Imager 600 (GE Healthcare, Little Chalfont, Buckinghamshire, UK) and densitometry using ImageJ^26^.

### Magnetic tweezers device

A custom-built magnetic tweezers apparatus was constructed around an Eclipse TE 2000-U inverted microscope (Nikon UK, Kingston-upon-Thames, Surrey, UK) with 100× 1.4 NA oil immersion objective lens and 100 W halogen lamp illumination, fitted with a custom-built two-axis piezo stage (SmarAct, GmbH, Oldenburg, Germany) and piezo objective focusing device (P721, PIFOC, Physik Instrumente, Cranfield, Beds UK). To increase the field of view, the camera was mounted on an additional 0.45 C-mount TV lens adaptor (Nikon). Two longitudinally magnetized, 5 mm diameter × 5 mm long cylindrical neodymium magnets (F641, MagnetExpert Ltd, Tuxford, Notts, UK) were mounted on a three axis manipulator with a custom-built, stepper-motor driven, rotation mount. The motor was coupled by a drive-belt system to avoid magnetic interference. Video images were usually recorded at 20 Hz with a gated, 10 ms exposure using a 1024 × 1280 CMOS sensor, Prosilica GC1280 GigE camera (Allied Vision, Stadtroda, Germany) and custom image capture software.

### Magnetic tweezers experiments

The microscope flow-cell was made by sandwiching two coverslips on top of one another held with double-sided sticky tape to give ~10 μl volume channels. The lower coverslip (nearest the objective lens) was previously cleaned with 5 M NaOH and treated with Vectabond reagent (Vector Laboratories, Burlingame, Ca, USA) to make it adhesive to proteins. Flow-cells were incubated with 100 µg/ml anti-digoxigenin antibody in PBS (AbD Serotec, Langford Lane, Kidlington, UK) at 37 °C for 2 h to coat the surface. The flow-cell was later incubated with the blocking solution (50 mg/ml polyacrylic acid (pH 7.0), 10 mg/ml BSA, 0.1% pluronic F127, 0.1 mg/ml casein) for 30 min at 23 °C. Streptavidin coated 1 μm MyOne beads (TFS) were mixed with the differentially end-labelled template DNA at an optimal ratio to ensure most of the beads were linked to the coverslip surface by a single DNA molecule (determined experimentally with each DNA batch). After 15 min incubation, the bead-DNA mix was subjected to the blocking solution for 30 min to passivate the bead surface and the mix was injected into the flow-cell and allowed to settle for 15 min. All unbound beads were washed out with 10–20 volumes (~70 μl) of working buffer, K100 + (100 mM Tris.HCl, pH 7.5, 100 mM KCl, 10 mM MgCl_2_, 1 mM EDTA, 0.2 mg/ml BSA). The flow-cell was used the same day. For the initial analysis of DNA fluctuations phosphate buffer was used (10 mM potassium phosphate, pH 8.0, 0.1% Tween-20, 0.1 mg/ml BSA). The stoichiometry of DNA-bead coupling was checked for every bead used in our data sets by supercoiling all beads positively and negatively at a force of 1.2–1.5 pN. Single DNA-bead tethers show a characteristic asymmetric z-dependence (see *Data analysis*, below) as negative supercoiling melts DNA. This does not happen when the bead is tethered by more than one DNA molecule because multiple DNA molecules become twisted around each other (braided) giving a very different z-dependence on supercoiling.

### Data analysis

Bead positions were tracked in three-dimensions using custom-written ImageJ^26^ macros. The x- and y-pixel dimensions were calibrated using a microscope reticule slide and the computed z-displacement was cross-calibrated against control images obtained by moving the microscope objective in a triangular waveform through a known distance (usually +/−2.5 μm) using the Pifoc focus device. The z-calibration was linear over a range of ~5 μm. z-displacement traces were filtered using a 25-point moving average. The vertical force, which extends the DNA molecule, generated by the magnetic tweezers was determined from the variance in x and y bead position^18^. Magnet height vs. bead force calibration graphs were obtained using video data acquired with a 2 ms shutter time on the CMOS camera in order to minimize motion blurring. Changes in bead height (due to nicking or DNA length fluctuations) were identified either directly by eye or by plotting histograms of the filtered z-displacement data and fitting the histograms to the sum of two Gaussian terms so that peak positions (mean DNA template lengths) and areas (time integrals) could be extracted from the raw data.

### Availability of materials and data

The materials and datasets generated during the current study are available from the corresponding author on reasonable request.

## Electronic supplementary material

Supplementary information
