# Supplementary material for: oriD structure controls RepD initiation during rolling-circle replication
Source: Sci Rep. 2018 Jan 19;8:1206. doi: 10.1038/s41598-017-18817-6 (PMC5775427; doi:10.1038/s41598-017-18817-6)
Supplement: Supplementary file 1 — Supplementary information [file 41598_2017_18817_MOESM1_ESM.pdf]

"*oriD structure controls RepD initiation during rolling-circle replication*"

Algirdas Toleikis[1,2], Martin R. Webb[1] and Justin E. Molloy[1*]

[1]The Francis Crick Institute, 1 Midland Road, London NW1 1AT, UK

[2]WMS - Cell and Development Biology, University of Warwick, Coventry, CV4 7AL, UK

Supplementary information

"*oriD structure controls RepD initiation during rolling-circle replication*"

Algirdas Toleikis[1,2], Martin R. Webb[1] and Justin E. Molloy[1*]

[1]The Francis Crick Institute, 1 Midland Road, London NW1 1AT, UK

[2]WMS - Cell and Development Biology, University of Warwick, Coventry, CV4 7AL, UK

Supplementary information

Relationship between DNA length and degree of supercoiling at low load:

As reported previously (18,20,21), dsDNA undergoes characteristic length changes when subjected to supercoiling by magnetic tweezers. At low load (<0.8 pN) and small amounts of supercoiling ($\sigma < \pm 0.2\%$) dsDNA deformation is mainly in the "twist" regime and the end-to-end length changes rather little. At higher levels of supercoiling, dsDNA undergoes writhe and shows dramatic shortening (**Fig. S1A**). Before every experimental run, all of the DNA-bead tethers in the field of view were subjected to a protocol of +/- 50 or +/- 20 magnet rotations (10-kb and 4-kb templates resp.) so that each molecule could be corrected for any thermally driven offset in superhelical density before the magnetic field was applied (**Fig. S1A**). The expected amplitude of torsional thermal fluctuation is given by the equipartition principle: $\frac{1}{2}k_bT = \frac{1}{2}\kappa(2\pi \mathrm{n})^2$; where $\kappa = (C/l_o)$, and at low longitudinal force (i.e. before the magnetic field is applied), C~160 pN.nm^2.rad^{-1} (19) and l_o= 3400 nm (10kb template) and 1360nm (4 kb template). This gives an expected r.m.s. angular deviation; $<\overline{\mathrm{n}}>$ = 1.47 and 0.93 turns for 10-kb and 4-kb templates resp. Our measured r.m.s. deviation in angular offset for the 10-kb template was 1.48 turns and for 4-kb, 1.18 turns. So, our measured variation is slightly larger than expected and this may be explained by additional rotational noise due to the random starting orientation of the paramagnetic bead easy axis relative to the magnetic tweezers which would contribute an additional (~0.25) turn variation. The reproducibility of the measurement was determined by paired replicates (**Fig. S1B**) which indicate the error for a given estimate was less than 0.5 turns (i.e. deviation from the unitary gradient indicated by the dotted line in **Fig. S1B**). The magnet rotation vs. z-displacement plots were also used to calculate the length change per turn of writhe (which was 69 ± 1.3 nm/turn over the range 8 to 32 turns, shown shaded on the plot) (**Fig. S1C**).

Supplementary **Figures S2-S3** are referred to in the main text.

Supplementary figures:

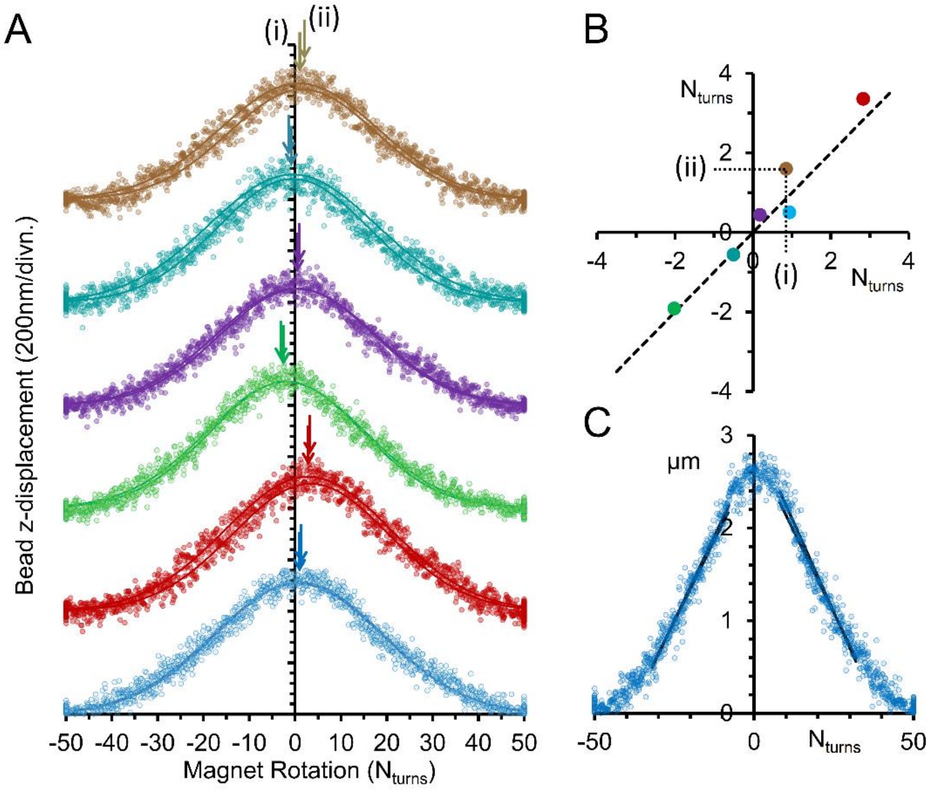

FIGURE S1. DNA tether length-dependence on supercoiling

In order to determine the tether length-dependence upon superhelical density, σ, the magnetic field was rotated by a known number of revolutions while recording bead height above the coverslip surface. Because the current study requires measurement of the absolute superhelical density we measured the initial offset due to thermal motion for each bead before the start of every experiment.

(A) The example shown (10-kb DNA tether, held at 0.4 pN, 23°C) illustrates the fact that DNA-bead constructs exhibit variable starting offset in super-helical density due to thermal rotation of the DNA-bead template before the magnetic field is applied to the sample. Six examples are shown, which were collected from a single field of view and data for each bead is plotted in a different color and offset with respect to the y-axis for clarity. The shape of the z-displacement vs rotation plots are used to determine the starting offset value; the data for each bead was fitted empirically to a Gaussian function (solid lines) and center values (indicated by arrows) were determined for two replicates for each bead. The fitted center values in the top record are labelled **(i)** and **(ii)** see **(B)** below. Raw data for the two experimental runs are overlaid in the figure.

(B) Reproducibility of the protocol is demonstrated by plotting the estimated center values (i.e. resting offsets due to rotational thermal motion before application of the magnetic field) for each bead for two replicates. The dotted line of unity gradient allows correlation between repeat measurements to be judged. The data (brown record) labelled **(i)** and **(ii)** in **(A)** represent the “worst case” and are shown for clarity as an example and show the largest deviation between the two replicates.

(C) A plot of z-displacement vs magnet rotation enables the relationship between length change (z-displacement) and turns of writhe to be determined. The gradient of the graph (solid lines) over the region (±) 8 to 32 turns (i.e. the linear, shaded, part of the plot) gives a length change of 69 ± 1.3 nm per turn of writhe (n=24).

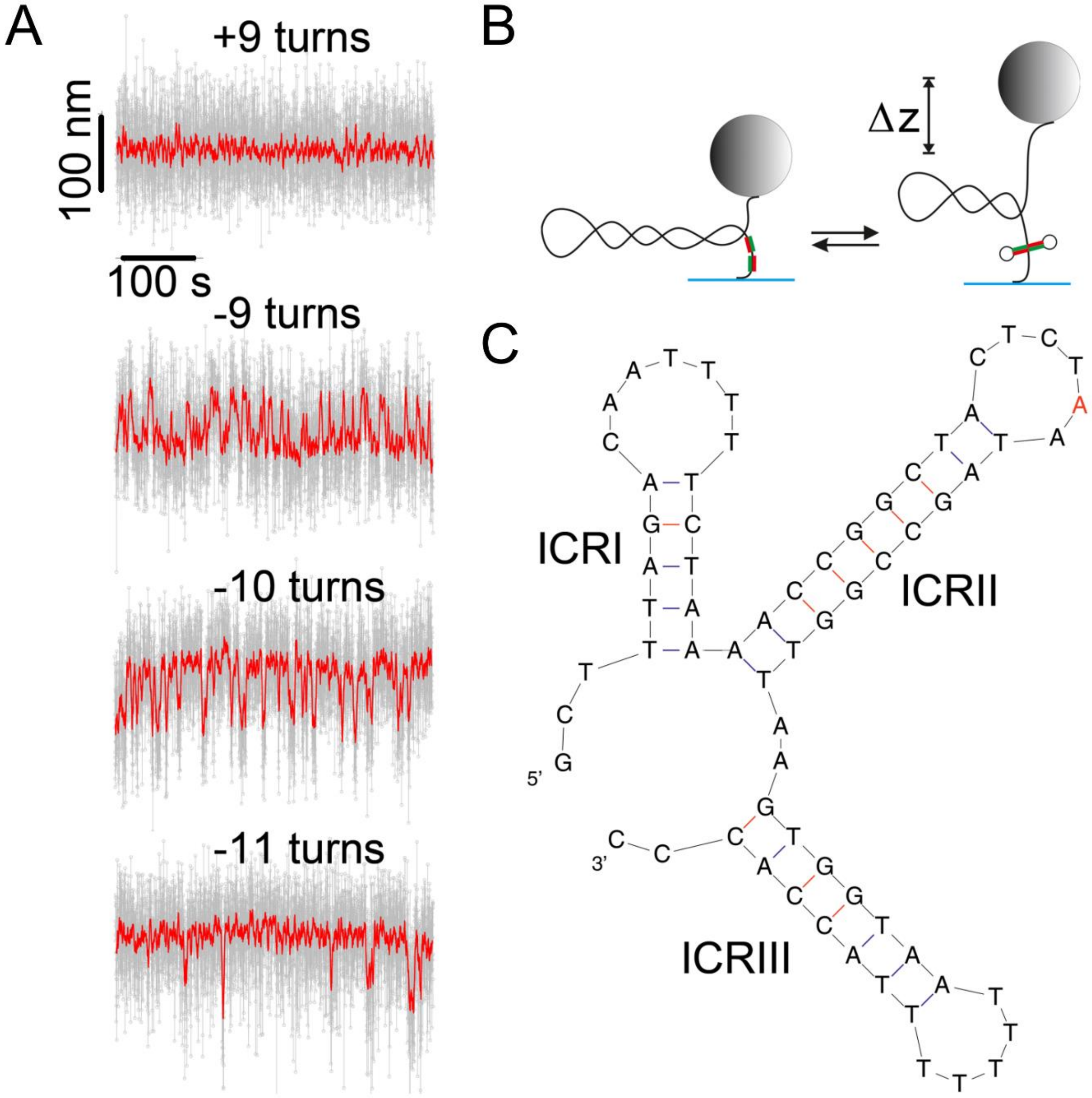

FIGURE S2. The *oriD*-containing DNA tethers fluctuate in length when negatively supercoiled.

(A) Length fluctuations were not observed when the DNA was positively supercoiled (e.g. upper trace +9 turns). Grey symbols are raw data; red lines are 25-point running averages. Experiments were conducted in the phosphate buffer (see Methods) without magnesium and at 26°C.

(B) Fluctuations in DNA tether length occur when ICRs interconvert between dsDNA backbone (B-state DNA) and hairpin structures.

(C) *oriD* consists of three ICRs, which can potentially form hairpin structures as illustrated.

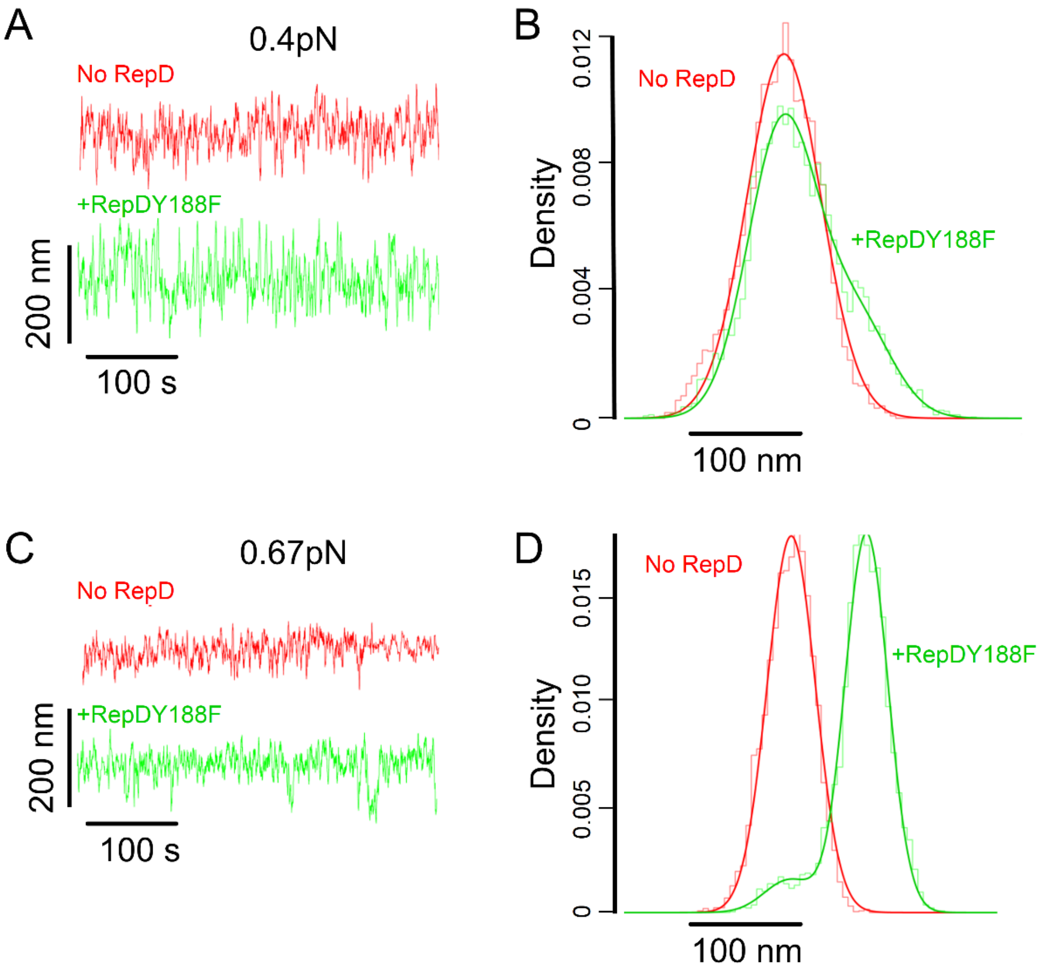

Figure S3. RepD stabilizes alternative DNA structures in *oriD*. Example time traces and method of analysis.

(A) DNA tether length fluctuations for a 4kbp DNA tether containing the wild type *oriD* sequence measured at 0.4 pN load and supercoiled to -10 turns in the absence (red) and presence of the non-nicking, RepD mutant protein RepDY188F (green).

(B) Histograms of length distributions for the corresponding data shown in panel (**A**). The smooth lines are least-squares fits to a dual-Gaussian function. $A_x = A_1 e^{\left(-\left(\frac{x-x_0}{<w>}\right)^2\right)} + A_2 e^{\left(-\left(\frac{x-(x_0+\delta x)}{<w>}\right)^2\right)}$ where the amplitude, A_1, is proportional to the fraction of time spent at the short tether length (x_0), A_2 is proportional to the fraction of time spent at the extended tether length ($x_0+\delta x$) where the length change δx = 71.5 nm (see main text **Fig. 7**) and the root mean squared deviation in DNA tether length ($<w>$) due to thermal motion is approximated by:

$$<w> = \sqrt{\frac{2L_0 L_p}{2 + (1 - L_{rel})^{-3}}}$$

Where DNA contour length, L_0 = 1360nm (4kbp) and persistence length, L_p = 50nm, the relative extension, L_{rel}, at a force of 0.4 pN, is 0.76: gives $<w>$ = 42 nm and at 0.67pN L_{rel} = 0.82: gives $<w>$ = 28 nm. The ratio of time spent in the two states (A_1/A_2) gives the equilibrium constants (tabulated in the main text **Fig. 6**).

(C&D) Panels are as for (**A&B**) but measured at 0.67 pN load.
